# Supplementary figures and images for: Regulation of transcription elongation anticipates alternative gene expression strategies across the cell cycle
Source: PLoS One. 2025 May 7;20(5):e0317650. doi: 10.1371/journal.pone.0317650 (PMC12057992; doi:10.1371/journal.pone.0317650)

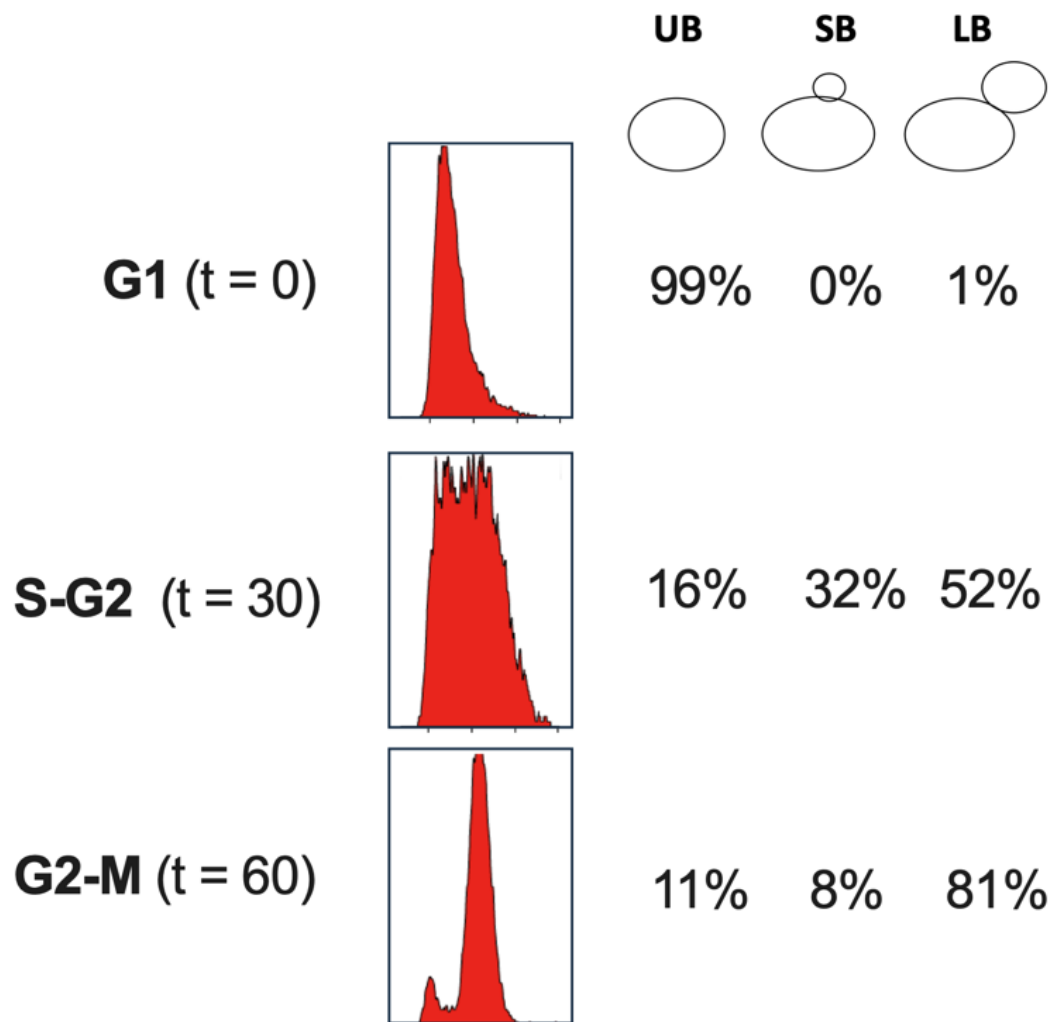

Supplement: S1 Fig — Representative example of the budding index and the FACS profile of samples in which stages in which active and total RNA pol II were analysed. (PDF) [file pone.0317650.s001.pdf]

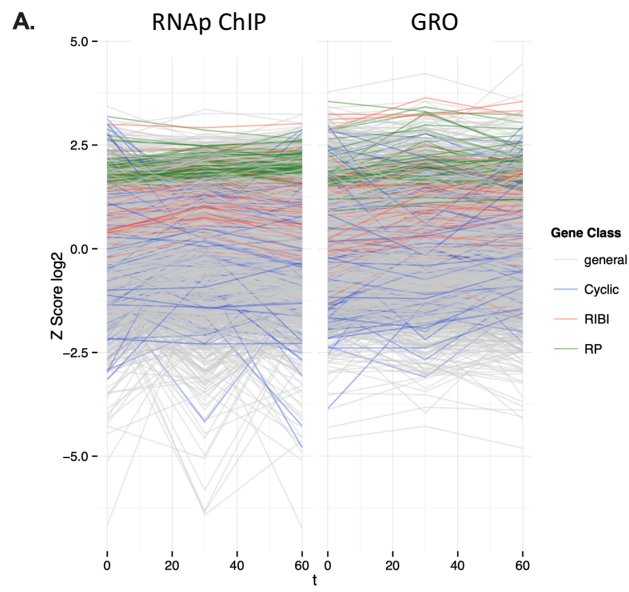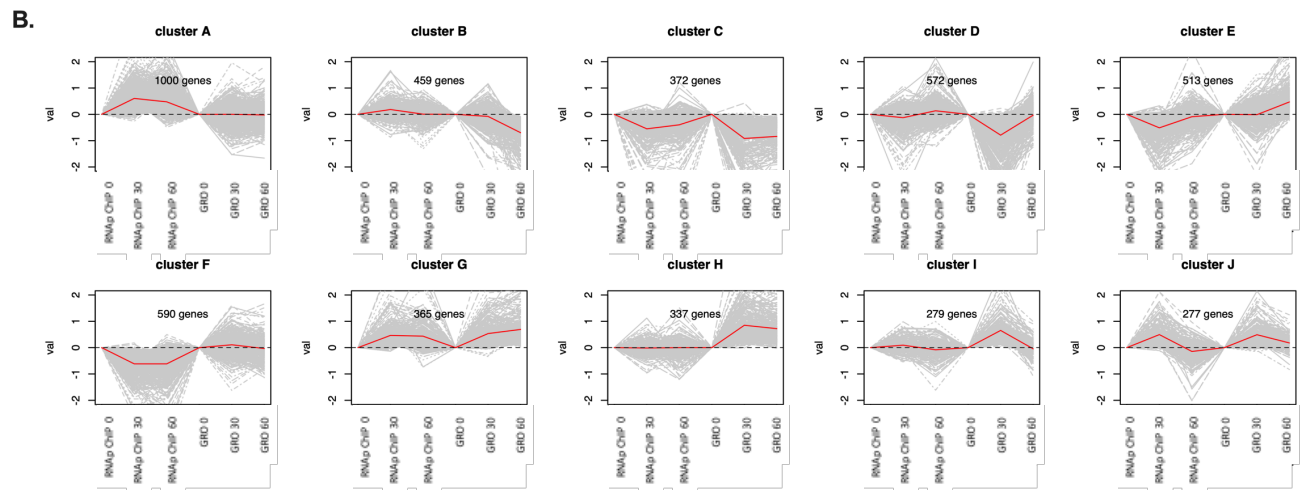

Supplement: S2 Fig — A) Plot representing total RNA pol II and active RNA pol II present in each gene at each time point analysed. B) Plot representing clusters identified with SOTA. (PDF) [file pone.0317650.s002.pdf]

A.

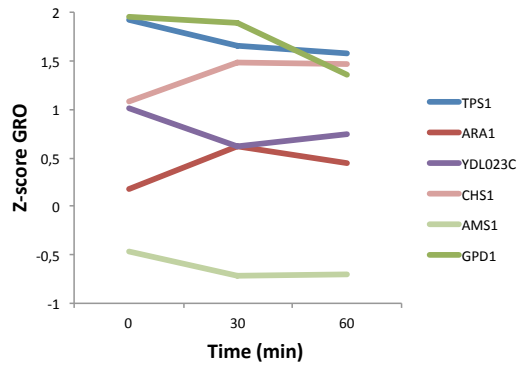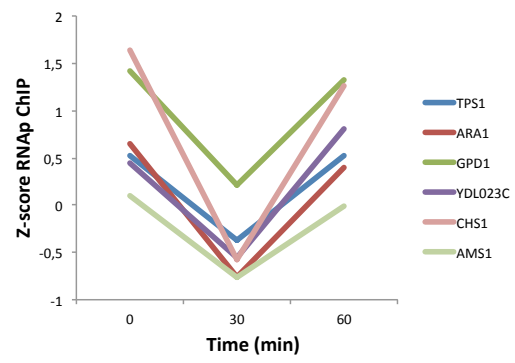

B.

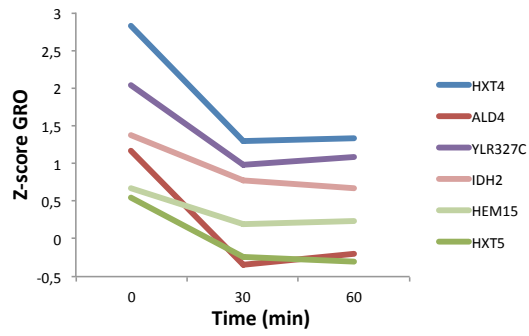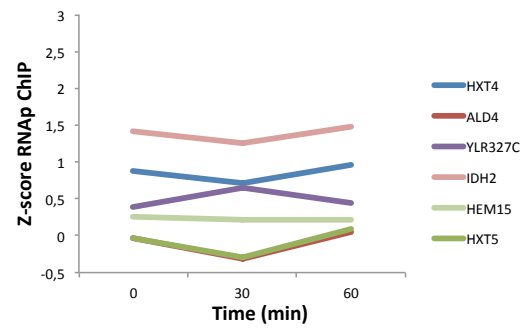

C.

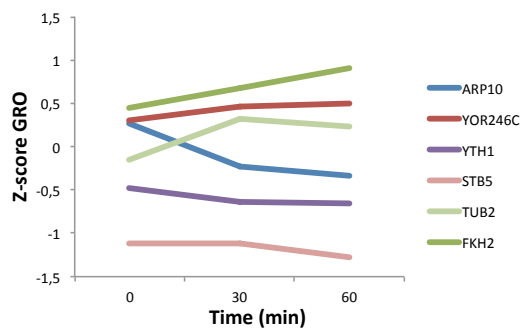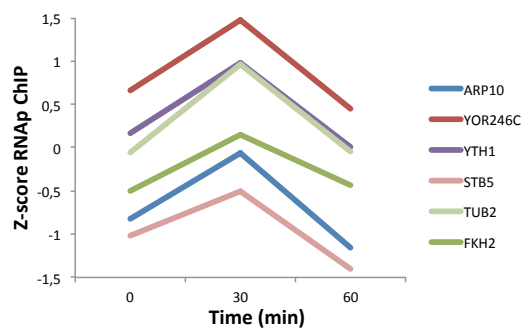

Supplement: S4 Fig — A) Examples of G1-expressed genes (AMS1, ARA1, CHS1, GPD1, TPS1 and YDL023C) belonging to cluster D. B) Examples of G1- (ALD4, HXT4 and HXT5) and G1/S-expressed genes (IDH2, HEM15 and YLR327C) from cluster F. C) Examples of S/G2- (ARP10) and G2/M-expressed genes (FKH2, STB5, TUB2, YOR246C and YTH1) from cluster I. (PDF) [file pone.0317650.s004.pdf]

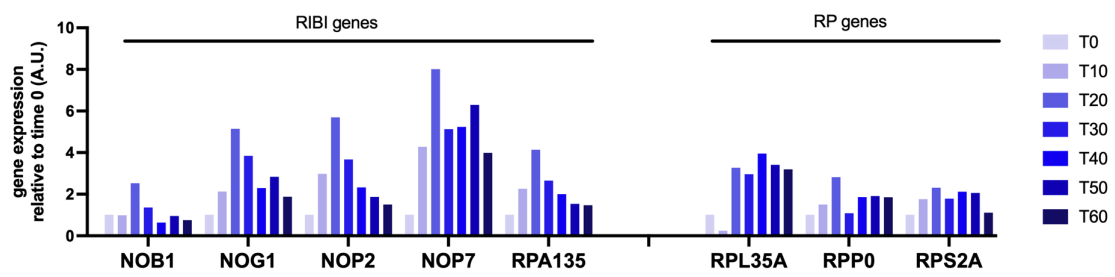

Supplement: S5 Fig — Gene ontology analysis highlights a strong enrichment of cluster A in RP and RIBI genes. Manual validation of mRNA levels obtained for selected RiBi and RP genes in synchronized cells released from a G1 arrest with alpha factor (10 minutes interval sampling). (PDF) [file pone.0317650.s005.pdf]
