# Supplementary material for: Regulation of transcription elongation anticipates alternative gene expression strategies across the cell cycle
Source: PLoS One. 2025 May 7;20(5):e0317650. doi: 10.1371/journal.pone.0317650 (PMC12057992; doi:10.1371/journal.pone.0317650)
Supplement: S3 Fig — Green arrows represent groups in which the enrichment or depletion of clusters is statistically significant (logistic regression with BF correction). (PDF) [file pone.0317650.s003.pdf]

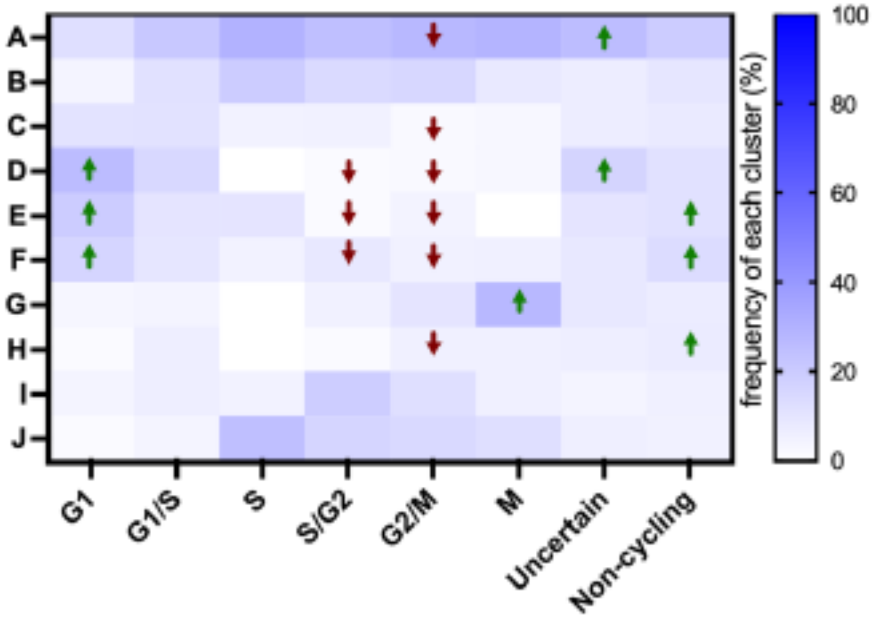
A
B
C
D
E
F
G
H
I
J
G1
G1/S
S
S/G2
G2/M
M
Uncertain
Non-cycling
frequency of each cluster (%)
100
80
60
40
20
0
